# Supplementary material for: Chromothripsis during telomere crisis is independent of NHEJ, and consistent with a replicative origin
Source: Genome Res. 2019 May;29(5):737–49. doi: 10.1101/gr.240705.118 (PMC6499312; doi:10.1101/gr.240705.118)
Supplement: Supplemental Material [file supp_gr.240705.118_Supplemental_file_1.zip › contigs/annotated_contigs/DB51/contig.2.DB51_length_285_mean_cov_5.47368421053.docx]

**DB51_length_285_mean_cov_5.47368421053**

```
GCAGTGGTTACCGTAACTATTTGTTCTCATATTTCTCCATCGTGGAAGCGAACAGCAGTATCCCAGTGATTTCCGTTTCTCTTACTCTG
 >chr20:2694740-2695004 + E=4e-136 p=0e+00
AGTGAGGTTGAGCCCACCAGGGCTATTTAAAGGCTATTTGTGTTTCCTTTACTGTGAACTGTCTGTTCATATCCTGTGCTTATTTTTCT

TTTCTCCTTCCTTCCTTCCTTCTTTCTTTCCTTCCTTCCTTCTTTCCTTCTCTCTTTCTCTCTCTCTTTCTTTCTTTTTTTT|CTTT|T
                                                                                   >chr
TTTTTTTTTTTTTTTTTGTG16:69163329-69163354
```
